# Wellbeing measures for workers: a systematic review and methodological quality appraisal

**DOI:** 10.3389/fpubh.2023.1053179

**Published:** 2023-05-24

**Authors:** Rebecca J. Jarden, Richard J. Siegert, Jane Koziol-McLain, Helena Bujalka, Margaret H. Sandham

**Affiliations:** ^1^Faculty of Medicine, Dentistry and Health Sciences, Melbourne School of Health Sciences, The University of Melbourne, Carlton, VIC, Australia; ^2^Austin Health, Heidelberg, VIC, Australia; ^3^Auckland University of Technology (AUT), North Shore Campus, Auckland, New Zealand; ^4^Department of Nursing, Melbourne School of Health Sciences, The University of Melbourne, Carlton, VIC, Australia

**Keywords:** employee wellbeing, psychometrics, quality appraisal, systematic review, work wellbeing

## Abstract

**Introduction:**

Increasing attention on workplace wellbeing and growth in workplace wellbeing interventions has highlighted the need to measure workers' wellbeing. This systematic review sought to identify the most valid and reliable published measure/s of wellbeing for workers developed between 2010 to 2020.

**Methods:**

Electronic databases Health and Psychosocial Instruments, APA PsycInfo, and Scopus were searched. Key search terms included variations of *[wellbeing OR “well-being”]* AND [employee^*^ OR worker^*^ *OR staff OR personnel]*. Studies and properties of wellbeing measures were then appraised using Consensus-based Standards for the selection of health Measurement Instruments.

**Results:**

Eighteen articles reported development of new wellbeing instruments and eleven undertook a psychometric validation of an existing wellbeing instrument in a specific country, language, or context. Generation and pilot testing of items for the 18 newly developed instruments were largely rated 'Inadequate'; only two were rated as 'Very Good'. None of the studies reported measurement properties of responsiveness, criterion validity, or content validity. The three instruments with the greatest number of positively rated measurement properties were the Personal Growth and Development Scale, The University of Tokyo Occupational Mental Health well-being 24 scale, and the Employee Well-being scale. However, none of these newly developed worker wellbeing instruments met the criteria for adequate instrument design.

**Discussion:**

This review provides researchers and clinicians a synthesis of information to help inform appropriate instrument selection in measurement of workers' wellbeing.

**Systematic review registration:**

https://www.crd.york.ac.uk/prospero/display_record.php?RecordID=79044, identifier: PROSPERO, CRD42018079044.

## 1. Introduction

Organizational interest in workers' wellbeing is increasing, and subsequently work wellbeing interventions are an area of growth. Wellbeing measures can both identify the need for an intervention through assessing the status of workers' wellbeing, and subsequently evaluate the efficacy of an intervention through quantifying the level of change in workers' wellbeing following the intervention. However, the numerous and growing number of available wellbeing measures [e.g., see (1, 2)], makes identifying and selecting the most appropriate, reliable, and valid instruments for effectiveness evaluations in the workplace difficult. Validity and reliability of these measures have not always been established and there is not yet a gold standard measure of wellbeing to evaluate the construct validity of new measures against. This review will inform future measurement development studies and improve clarity for researchers and clinicians in instrument selection in the measurement of workers' wellbeing.

### 1.1. Work wellbeing

Theoretical models and definitions of work wellbeing are varied and usually from a Western perspective (3–5). The construct of workers' wellbeing is rich and multifaceted, scaffolding elements that transcend work (the role), workers (the individuals and teams) and workplaces (organizations) (6). Key factors are thought to include subjective wellbeing, including job satisfaction, attitudes and affect; eudiamonic wellbeing including engagement, meaning, growth, intrinsic motivation and calling; and social wellbeing such as quality connections and satisfaction with co-workers (7). Laine and Rinne (8) add to these factors in their “discursive” definition which encompasses healthy living/working, work/family roles, leadership/management styles, human relations/social factors, work-related factors, working life uncertainties and personality/individual factors. Work-Related Quality of Life (WRQoL) add further factors, including general wellbeing, home-work interface, job and career satisfaction, control at work, working conditions and stress at work (9).

The elements associated with wellbeing differ between occupational groups (10). For professionals, five elements typically account for the greatest amount of variance in job satisfaction: work-life balance, satisfaction with education, being engaged, experiencing meaning and purpose, and experiencing autonomy (10). Knowing what constructs workers find meaningful with respect to wellbeing determines the essential content in a wellbeing measure and can vary between occupational groups. For example, laborers value work-life balance, being absorbed, meaning and purpose, feeling respected and having self-esteem (10), whereas nurses valued workplace characteristics, the ability to cope with changing demands and feedback loops (11).

### 1.2. Measuring workers' wellbeing

Given the variations in theoretical models, definitions of, and salience of elements associated with wellbeing in different occupational groups, selecting instruments for the measurement of workers' wellbeing is challenging. While there are multiple methodologies for investigating workers' wellbeing, in this review we focus on quantitative assessment. Two directions in the measurement of workers wellbeing have been taken. First, to use existing wellbeing instruments with workers. Second, to develop new instruments specifically intended to measure workers' wellbeing. The decision to use a given workers' wellbeing measure may be guided by many factors, but it is essential to prioritize the measurement properties of the instrument, such as the reliability, validity and responsiveness of the instrument (12). A single “gold-standard” measure of workers' wellbeing has not yet been identified and given the afore mentioned heterogeneity in the construct of wellbeing depending on the viewer, a one size fits all gold standard is unlikely to be found. The most appropriate instrument to measure the construct may require a selection of unidimensional (sub) scales, like the measurement of WRQoL (9). For this review, the aim was to evaluate the measurement properties of instruments that measured the broader construct of workers' wellbeing [e.g., the Workplace Wellbeing Index (13, 14)]. Any identifiable sub-scales within the instruments were individually reported.

### 1.3. Systematic reviews of measurement instruments

The systematic review is one method of identifying, appraising and synthesizing research to strengthen the evidence base and inform decisions. The Preferred Reporting Items for Systematic review and Meta-Analysis (PRISMA) guidelines (15) supports both rigor and transparency in reviews. A systematic review of studies developing, and reporting, instrument measurement properties enables the generation of new evidence, in much the same way as a systematic review of clinical studies or trials is essential for establishing the effectiveness of an intervention. Well-defined criteria for appraising the methodological quality of studies of instrument measurement properties are therefore important for establishing evidence for the measurement properties of instruments. One such methodology to support this appraisal process was developed through the international COnsensus-based Standards for the selection of health Measurement Instruments (COSMIN) initiative (https://www.cosmin.nl/) which sought to improve the selection of outcome measurement instruments for both research and clinical practice [e.g., see (12, 16–24)].

### 1.4. Systematic reviews of wellbeing measures

There were four previous reviews of measures for assessing wellbeing in adults identified (2, 25–27). McDowell (27) reviewed nine specifically selected measures reported by the author to be representative of different conceptualizations of wellbeing. These measures were all developed before 2000 and included: Life Satisfaction Index, the Bradburn Affect Balance Scale, single-item measures, the Philadelphia Morale Scale, the General Wellbeing Schedule, the Satisfaction With Life Scale, the Positive and Negative Affect Scale, the World Health Organization 5-item wellbeing index, and the Ryff's scales of psychological wellbeing. McDowell (27) described the nine measures and their properties. Lindert et al. (26) aimed to identify, map and analyze the contents of self-reported wellbeing measurement scales from studies published between 2007 and 2012. Sixty measures were identified, described, and appraised using an author developed evaluation tool based on the recommendations of the Scientific Advisory Committee of the Medical Outcomes Trust and two checklists for health status instruments (28–31). Linton et al. (2) reviewed 99 self-report measures from studies published between 1993 to 2015 for assessing wellbeing in adults, exploring dimensions of wellbeing and describing development over time using thematic analysis and narrative synthesis. Ong et al. (25) conducted a broad scoping review to identify measures to assess subjective wellbeing, particularly in the online context, using thematic coding. None of these four reviews used the COSMIN methodology or focused specifically on the wellbeing of workers.

### 1.5. Objectives

This review aims to: (1) systematically identify articles published from 2010 to 2020 reporting the development of instruments to measure workers' wellbeing, (2) critically appraise the methodological quality of the *studies* reporting the development of workers' wellbeing measures, (3) critically appraise the psychometric properties of the *measures* developed for workers' wellbeing, and (4) based on the measures developed between 2010 and 2020, recommend valid and reliable measures of workers' wellbeing. As such, this review informs future measurement development studies and improves clarity for researchers and clinicians in instrument selection in the measurement of workers' wellbeing.

## 2. Methods

This systematic review largely followed the methods published in the review protocol (32). Four review protocol variations were required.

### 2.1. Review protocol variations

The four protocol variations were needed due to project scope and feasibility, new reporting standards being developed between publication of the protocol and completing the review (15), improved access to programs (e.g., Covidence, Veritas Health Innovation Ltd), updated versions of programs (e.g., Endnote X9), and evolving knowledge of databases, wellbeing definitions, and terminology (in consultation with liaison research librarians across two universities). First, project scope and feasibility were managed through limiting the databases searched to Health and Psychosocial Instruments, APA PsycInfo, and Scopus. These three databases were selected in consultation with a research librarian to maintain breadth. We included a manual reference list review and forward and backward citation chaining of potentially relevant reviews and included studies to strengthen the search. Second, the article publication date range of 2010 to 2020 was applied as a limiter to manage project scope and was selected to align with publication of the COSMIN checklist [e.g., see (16–19)], building on earlier work [e.g., (33)]. Third, we have used the updated a Preferred Reporting Items for Systematic review and Meta-Analysis (PRISMA) guidelines (15) and COSMIN methodology (19–21, 23, 24, 34). Fourth, latest versions of Endnote (X9) citation management software and Covidence review management software (Veritas Health Innovation Ltd) were used to support the review processes.

### 2.2. Review inclusion and exclusion criteria

#### 2.2.1. Types of instruments

Eligible workers' wellbeing data collection instruments included interviewer-administered, self-administered or computer-administered. Examples included an online survey, a written questionnaire completed by a worker, or a worker's responses to an interviewer administering the survey.

#### 2.2.2. Types of study design

Eligible studies were those published as a full text original article that report psychometric properties and (1) development of an entirely new instrument or (2) validation of an instrument modified from a previously developed instrument.

#### 2.2.3. Types of settings and participants

The study sample needed to include workers. If other populations were included along with workers, the findings related to workers needed to be differentiated from others. The measure could have been applied to workers in any paid work setting where a workplace is defined as a place where a worker goes to carry out work (35). For articles reporting multiple studies using several different samples, only those that included workers in at least one sample were included.

#### 2.2.4. Types of measures

Instruments developed or validated for the measurement of workers' wellbeing as an outcome were eligible for inclusion. The disparate theoretical views and definitions of both wellbeing (36–38) and work wellbeing (3–5, 8, 39) lead us to include instruments where the term “wellbeing” was specifically stated as either “wellbeing,” “well-being” or “well being.” The term “workers”' needed to be specifically stated as either “employee^*^,” “worker^*^,” “staff” or “personnel.” Studies reporting the use of instruments to measure commonly cited terms for high levels of wellbeing including flourishing (40, 41) and thriving (42–44) were included. Studies in which authors stated they were developing or validating a measure of workers' wellbeing, but only used items or previously developed instruments of other constructs (e.g., happiness, or positive and negative emotions, or satisfaction with life, or depression, or stress or anxiety) were excluded. Studies and measures published in languages other than English were excluded. Abstracts, books, theses and conference proceedings were excluded.

### 2.3. Search strategy

A three-staged search strategy was used to identify studies that include measures meeting the inclusion criteria: (1) electronic bibliographic databases for published work, (2) reference lists of studies with included measures, and (3) the reference list of previously published reviews.

#### 2.3.1. Information sources

The following electronic bibliographic databases were searched: Health and Psychosocial Instruments (abstract search), APA PsycInfo (abstract search), and Scopus (title, abstract & keyword search).

#### 2.3.2. Search terms

Database key search terms included *[wellbeing OR “well-being”]* AND [employee^*^ OR worker^*^ *OR staff OR personnel]*. Search terms for measurement properties of measurement instruments were adapted from the “precise search filter for measurement properties” and “exclusion filter” (45). The search strategy is provided in Supplementary material.

### 2.4. Data management

References identified in execution of the search strategy were exported to EndNote X9 bibliographic software, and duplicates were removed. References were imported to Covidence, Veritas Health Innovation Ltd for duplicate screening, appraisals and data extraction.

### 2.5. Selection process

Titles and abstracts were screened by two independent reviewers (RJ or MS or SB or JD). The full text documents of these potentially relevant studies were independently screened against the eligibility criteria by two reviewers (RJ or MS and SB or JD). Any disagreement was resolved through consensus amongst the review team. Findings from the execution of the search and selection process are presented in a Preferred Reporting Items for Systematic review and Meta-Analysis (PRISMA) flowchart (15).

### 2.6. Data collection process

Data were extracted by two reviewers independently (RJ and/or MS and/or HB) into Covidence 2.0 templates adopted from the COSMIN methodology user guide (20). Final data tables were checked for accuracy and completeness by a third reviewer (RJ and /or MS and /or HB).

### 2.7. Data analysis process

The findings from execution of the search strategy are described and illustrated in a flow chart; the characteristics of the included studies are tabulated. Analysis of methodological quality followed the procedure of COSMIN methodology for systematic reviews of Patient-Reported Outcome Measures (20) and supporting resources (19–21, 23, 24, 34). The COSMIN Risk of Bias assessment distinguish between appraisal of content validity, that is the extent to which the area of interest is comprehensively addressed by the items in the instrument, and appraisal of the process of measurement instrument development (24). Although appropriate instrument design studies support good content validity, distinct appraisal criteria should be applied to instrument development studies and to studies that assess content validity of existing measurement instruments. In the present review, two reviewers (RJ and/or HB and/or MS) independently appraised studies that developed new wellbeing instruments against the COSMIN Patient-Reported Outcome Measure (PROM) development criteria (19, 20, 23), and appraised studies that validated existing instruments against the COSMIN content validity criteria (23). The term “Patient” in “PROMs” is considered synonymous with the population group for this study, “Worker.” Reviewer consensus occurred through discussion.

#### 2.7.1. Assessment of the methodological quality of the included studies

The COSMIN checklist includes 10 boxes: two for content validity, three for internal structure, and five for the remaining measurement properties of reliability, measurement error, criterion validity, hypotheses testing for construct validity and responsiveness (19, 20). Studies were rated as either “Very Good,” “Adequate,” “Doubtful,” or “Inadequate.” The rating “Not Explored” was applied for any measurement properties not investigated for an instrument in any individual article. We have briefly summarized key criteria below based on the COSMIN taxonomy, for further detail please see associated COSMIN methodology user manuals and reference materials (19–21, 23, 34).

##### 2.7.1.1. Structural validity, internal consistency and measurement invariance

Three measurement properties relate to the internal structure of an instrument: structural validity, internal consistency, and measurement invariance. Structural validity can be assessed for multi-item instruments that are based on a reflective model where each item in the instrument (or subscale within an instrument) reflect an underlying construct (for example, psychological wellbeing) and should thus be correlated with each other (20). For the methodological quality of studies of structural validity to be rated “Very Good,” the study must conduct confirmatory factor analysis; include an adequate sample size with respect to the number of items in the instrument; and not have other methodological flaws. Internal consistency is the degree to which items within an instrument (for a unidimensional instrument) or subscale of an instrument (for a multidimensional instrument) are intercorrelated with each other. For studies of internal consistency, the COSMIN Risk of Bias checklist stipulates that a rating of “Very Good” requires the study of internal consistency to report the Cronbach's alpha (or omega) statistic (and for each subscale within a multi-dimensional scale), and for no other major methodological or design flaws in the study. Measurement invariance (also known as cross cultural validity) is the extent to which the translated or culturally modified version of an instrument perform in a similar way to those in the original version. A rating of “Very Good” for the methodological quality of studies of measurement invariance requires evidence that samples being compared for different versions of the instrument are sufficiently similar in terms of any relevant characteristics (except for the key variable that differs between them, such as cultural context); that an appropriate method was used to analyze the data (for example, multi-group confirmatory factor analysis); and there is an adequate sample size, which is dependent on the number of items in the instrument of interest (19–21, 23).

##### 2.7.1.2. Reliability

Reliability is the proportion of variance in a measure that reflects true differences between people and is assessed in test-retest studies; to avoid confusion with other forms of reliability, it is hereafter referred to as test-retest reliability. For the methodological quality of a test-retest reliability study to be rated as “Very Good,” it must provide evidence that respondents were stable between repeated administration of the test instrument; the interval separating repeated administration of the instrument must be appropriate; and the study must provide evidence that the test conditions between repeated tests were similar. Regarding the statistical methods, the COSMIN Risk of Bias tool specifies that for continuous scores the intraclass correlation coefficient must be calculated (19–21, 23).

##### 2.7.1.3. Measurement error

Measurement error refers to the error, whether systematic or random, in an individual's score that occurs for reasons other than changes in the construct of interest. Similar to studies of test-retest reliability, for studies of measurement error to be rated as “Very Good,” evidence must be provided that respondents were stable between repeated administration of the instrument, that the interval between repeated administrations of the instrument were appropriate, and that the test conditions were similar for repeated administrations of the instrument. Regarding the appropriateness of statistical methods, standard error of measurement (SEM) or smallest detectable change (SDC) must be reported for continuous scores (19–21, 23).

##### 2.7.1.4. Criterion validity

Criterion validity is the extent to which scores on a given instrument adequately reflect scores of a “gold standard” instrument that assesses the same construct. For the methodological quality of a study of criterion validity to be rated as “Very Good,” correlations between the instruments must be reported for continuous scores, and the study must be free from other methodological flaws (19–21, 23). For workers' wellbeing, our systematic search of the literature did not identify a universally accepted “gold standard” for workers' wellbeing for use in evaluating criterion validity. However, given the varied definitions and models of wellbeing, we have evaluated criterion validity for included studies. We have based our evaluation on the individual study authors' definition or model of workers' wellbeing and it's alignment to their selected “gold standard” instrument.

##### 2.7.1.5. Construct validity

Construct validity is the extent to which scores on an instrument are consistent with hypotheses about the construct that it purports to measure. Two broad approaches to hypothesis testing for construct validity are the “convergent validity” approach and the “discriminative or known-groups validity” approach. Hypothesis testing for convergent validity involves comparison on performance on the instrument of interest and another instrument that measures a construct that is hypothesized to be related or unrelated in some way. For studies employing the convergent validity approach to establishing construct validity to be methodologically rated as “Very Good,” the construct measured by the comparator instrument must be clear; sufficient measurement properties of the comparator instrument must have been established in a similar population and the statistical methods must be appropriate. For studies employing the “discriminative or known groups validity” approach to establishing construct validity to be methodologically rated as “Very Good,” the study must adequately describe the relevant features of the subgroups being compared, and appropriate statistical methods must be employed (19–21, 23).

##### 2.7.1.6. Responsiveness

The measurement property of responsiveness refers to the ability of an instrument to measure changes over time in the construct of interest. It is similar to construct validity, but whereas construct validity refers to a single score, responsiveness refers to the validity of a change in the score, for example, the ability of the instrument to detect a clinically important change. The COSMIN Risk of Bias tool provides standards for assessing the methodological quality of numerous subtypes of responsiveness; for example, for the methodological quality to be rated “Very Good” for a study using the “construct” approach to responsiveness, the study must adequately describe the intervention, and use appropriate statistical methods (19–21, 23).

The COSMIN guidelines recommend that if PROM development studies or content validity studies are rated as “Inadequate,” then measurement properties should not be assessed. However, we determined that we would appraise the qualities of studies on other measurement properties, even if the initial PROM development was rated as “Inadequate.” By continuing with these further assessments and providing readers with the detailed findings of these assessments, our review will enhance opportunities to strengthen future workers' wellbeing measure development.

##### 2.7.1.7. Evaluation of the study results against criteria for good measurement properties

The quality of the measurement instruments was rated as either “Sufficient,” “Insufficient,” or “Indeterminate” against the criteria of good measurement properties (21). Briefly, the criteria for a rating of “Sufficient” for each of the measurement properties are as follows; for further detail, see Prinsen et al. (21). For structural validity, the model fit parameters of a confirmatory factor analysis must meet specified criteria. For internal consistency, an instrument must have at least low level of sufficient structural validity and Cronbach's alpha must be ≥0.7. Thus, in the case that there is “Insufficient” structural validity (for example if structural validity assessment was undertaken only with exploratory factor analysis), internal consistency cannot be appraised even if it has been calculated and reported. For test-retest reliability, the intraclass correlation coefficient must be ≥0.7. For measurement error, the minimally important change (MIC) must exceed smallest detectable change (SDC); whereas the SDC is the smallest change that is attributable to measurement error, the MIC is the smallest change that can be detected that respondents perceive as important. For an instrument's construct validity, the results of hypothesis testing for construct validity must be supported. For measurement invariance, there must be no important differences in the model between the groups being compared. For criterion validity of an instrument, correlation with a “gold standard” must be ≥0.7. For responsiveness, the results of a study of responsiveness must support the hypothesis. We also appraised interpretability, or the extent to which one can assign qualitative meaning to a quantitative score (21, 33). However, diverging from the recommendations of Terwee et al. (33) and subsequently Prinsen et al. (21) we applied a two-category scoring system to assess interpretability, with a positive rating for studies that report at least some descriptive statistics for the instrument for the sample of interest, and a negative score for studies that did not report descriptive statistics. This differs somewhat to the recommendations of Terwee et al. (33), who recommend that the minimally important change (MIC) must be reported for a favorable rating of interpretability.

### 2.8. Data synthesis

The inconsistency in individual study populations, settings and languages did not support meta-analysis, statistical pooling nor a cumulative evidence grade [see De Vet (12) for further information]. Rather, results are tabulated with statistical summaries and a narrative description.

## 3. Results

**Figure 1:**
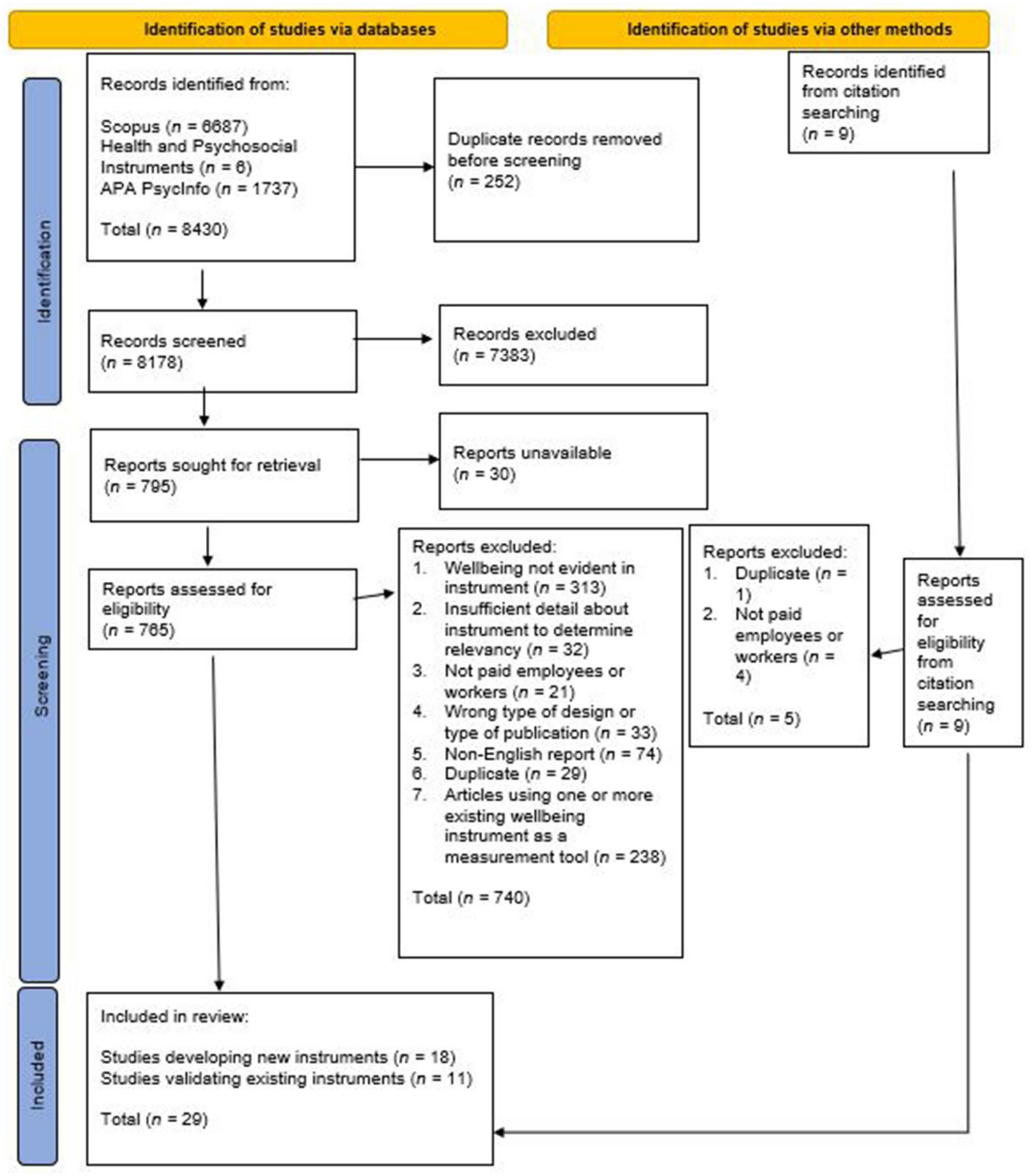
Search and screening flow diagram. Flow diagram adapted from Page et al. (15).

The initial search returned 8,430 articles; once 252 duplicates were removed, the titles and abstracts of 8,178 studies were screened for relevance, resulting in removal of 7,383 irrelevant studies. Of the remaining 765 full-text studies that were assessed for eligibility, 502 were excluded for reasons including wellbeing not being evident in the instrument (e.g., the instrument measured stress, anxiety, depression); the wrong type of publication (e.g., qualitative research not using a measurement instrument, non-primary research); and insufficient detail about the instrument to determine relevance. Citation searching returned nine further studies for screening (see Figure 1).

Data were extracted from the remaining 267 articles, to identify: (1) articles that reported the development of a new instrument (which may or may not include workers in the development stage) and that also psychometrically validate it in a sample of workers/employees; (2) articles that reported, as the primary aim of the study, a psychometric validation—in a specific country, language, or context—of an existing work wellbeing instrument that was originally developed more than 10 years ago and/or in a different context or population. Articles that reported the use of one or more existing wellbeing instruments for the purpose of measuring wellbeing as an outcome, rather than reporting instrument development or measurement properties, were excluded at this point (*n* = 238). The following analysis and results are for the articles that report the development of a new wellbeing instrument (*n* = 18) and those that psychometrically validate a previously developed wellbeing instrument in a new population, language, culture, or context (*n* = 11). Within each of these two groups, we appraised both methodological quality of the *studies* of instrument measurement properties, and the psychometric properties of the *instruments*.

### 3.1. Characteristics of articles reporting development of new instruments

The 18 articles that report the development of a new instrument, and the identified psychometric properties, are summarized in Table 1.

**Table 1:** Characteristics of articles reporting development of new instruments.

**References**	**Instrument**	**Instrument details**	**Study population**	**Demographics**	**Validity**	**Internal**	**Test-retest**	**Measurement**
**Country/**	**name**	**(scales, factors**,	***N***	**Age mean (SD)**	**parameters**	**consistency**	**reliability**	**error**
**language of**	**Brief description**	**etc)**				**(α)**		
**study**	**of instrument**	**Response format**						
**Aim**				**% Female**				
Anderson et al. (46)	Personal Growth and Development Scale (PGDS)	15-item, 5-factor scale with 3 items per factor	Full-time and part-time workers	Age: 36.90 (10.98)	Structural	0.96	NE	NE
Country not specified/English		7-point response scale, 1 = not at all, 7 = very much so	*n =* 468	% Female: 46.58%	Construct			
To develop and validate a context-specific measure of personal growth and development	A context-specific measure of personal growth and development				Cross-cultural validity/measurement invariance			
Butler and Kern (47)	PERMA-Profiler	A 23-item scale with 5 factors	Employees of a creative online organization from 24 teams from five global offices	Age: Mean and SD not reported	NE^#^	Overall PERMA Profiler: 0.84 Positive emotion sub-scale: 0.69 Engagement sub-scale: 0.77 Relationships sub-scale: 0.89 Meaning sub-scale: 0.70 Accomplishment sub-scale: 0.92	NE	NE
Country and language not specified		11-point Likert scale with 0 indicating extremely low levels and 10 indicating extremely high levels	*n =* 294					
To develop and validate the PERMA Profiler	A brief measure of wellbeing based on the five domains defined by Seligman's PERMA theory: positive emotion (P), engagement (E), relationships (R), meaning (M), and accomplishment (A)			% Female: 48.98%				
Chung et al. (48)	Nursing Health and Job Satisfaction (NHJS) scale	3 subscales: Wellbeing subscale Work environment satisfaction subscale Health Promoting lifestyle subscale	Registered nurses in a Taiwanese medical center	Age: NR	Structural	Wellbeing: 0.91 Work-Environment Satisfaction: 0.91 Health-Promoting Lifestyle: 0.83	NE	NE
Taiwan/Chinese	Scales to measure nurses' wellbeing, health-promoting lifestyle, and work environment satisfaction (WHS)		*n =* 672					
To develop and validate measurement scales and a conceptual model of nurses' wellbeing, health-promoting lifestyle, and work environment satisfaction		5-point Likert scale		% Female: NR				
Dagenais-Desmarais and Savoie (49)	Index of Psychological Wellbeing at Work (IPWBW)	25 items; 5 dimensions: Interpersonal Fit at Work Thriving at Work Feeling of Competency at Work Desire for Involvement at Work Perceive Recognition at Work	Québec workers recruited mainly from professional corporations representing paramedical, administrative, and scientific sectors	Age: 37.7 (10.2)	Structural	IPWBW Instrument: 0.96	NE	NE
Canada/French	An instrument for measuring psychological wellbeing at work as described by workers themselves				Construct	Dimensions: Interpersonal Fit at Work: 0.92 Thriving at Work: 0.91 Feeling of Competency at Work: 0.86 Desire for Involvement at Work: 0.89 Perceived Recognition at Work: 0.83		
To develop an inductive model for psychological wellbeing at work (PWBW) starting from workers' experiences and then submit it to empirical verification		6-point Likert scale (0 = Disagree, 6 = completely agree)	*n =* 1,080	% Female: 75.60%				
den Kamp et al. (50)	Proactive vitality management (PVM) scale	8-item, 1-factor scale	Employees in a wide range of professions and industries	Age Study 1: 34.98 (13.24) Study 2: 36.26 (10.57) Study 3: 36.43 (12.96)	Structural	PVM Scale: 0.86	NE	NE
Netherlands/Dutch	A measurement instrument that captures the proactive behavioral component (i.e., self-initiated and goal-oriented behavior) and both the physical and mental aspect of vitality	7-point response scale (totally agree to totally disagree)	Study 1: *n =* 813 Study 2: *n =* 113 Study 3: *n =* 246		Construct			
To develop and validate a reliable measurement instrument to capture people's proactive vitality to promote their work.				% Female Study 1: 43.4% Study 2: 48% Study 3: 48.4%				
Eaton et al. (51)	Work-related wellbeing (WRWB) index	11-item, 3-factor scale	Full- and part-time, permanent Federal employees from 37 federal agencies, including small, medium, and large organizations	Age	Structural	WRWB Instrument: 0.88	NE	NE
USA/English	An index based on the principles of positive psychology and subjective wellbeing that assesses the social and psychological components of respondents' attitudes toward their job	Five-point Likert response scale	*n =* 392,752	NR	Construct	Subscales: Work positivity: 0.80 Work mastery: 0.78 Co-worker relationships: 0.80		
To describe development and validation of the work-related wellbeing (WRWB) index				% Female: 48.10%				
Juniper et al. (52)	Work and Wellbeing Assessment for Libraries	42-item, 8-domain scale	Employees within the Hampshire County Council Library and Information Service (LIS) public service based in Southern England	NR	NR	Subscales: Organizational: 0.92 Advancement: 0.85 Job domain: 0.85 Physical Health: 0.81 Psychological Health: 0.74 Interpersonal Relationships: 0.85 Workload: 0.84 Facilities: 0.74	NE	NE
UK/English	A questionnaire to determine the ways in which working in a UK public library system can impact the wellbeing of those deployed in the sector	5-point scale (1 = not at all important and 5 = extremely important)	*n =* 466					
To develop and pilot a questionnaire to determine the ways in which working in a UK public library system can impact the wellbeing of those deployed in the sector								
Kazemi (53)	Occupational Social Wellbeing Inventory (OSWI)	20-item, 5-factor scale	Teachers, principals, and other members of staff at six different schools (at the levels of preschool, primary and secondary school) in a small Swedish municipality	Age: NR	Structural^*^	Subscales: Social integration: 0.91 Social acceptance: 0.83 Social coherence: 0.63 Social actualization: 0.64 Social contribution: 0.57	NE	NE
Sweden/Swedish	A measure of multi-dimensional view of occupational social wellbeing	A seven-point response scale	*n =* 314	% Female: 76.10%	Construct			
To investigate the validity of a multi-dimensional view of occupational social wellbeing								
Kern et al. (54)	A scale based on Seligman's multidimensional PERMA (positive emotion, engagement, relationships, meaning, and accomplishment) model of flourishing	36-item, six-factor scale	Employees from a large private school in Australia	Age: NR	Structural	Factors: Positive Emotion: 0.86 Engagement: 0.89 Relationships: 0.97 Meaning: 0.89 Accomplishment: 0.82 Negative Emotions: 0.87	NE	NE
Australia/English			*n =* 153					
To investigate the effects of a multidimensional measure of educational staff wellbeing on physical health, life satisfaction, job satisfaction, and organizational commitment				% Female: 48%				
Khatri and Gupta (55)	Employee wellbeing scale	23-item, 4-dimension scale	Professionals working in the IT/ITES and BFSI organizations based in Delhi-NCR	Age	Structural	Dimensions: Work-life Balance: 0.86 Job wellness: 0.82 Purpose in life: 0.80 Physical wellness: 0.71	NE	NE
India/not specified	A measure for the employee wellbeing construct comprised of four dimensions namely, purpose in life, work–life balance, job wellness, and physical wellness	5-point Likert response 1 (strongly disagree) to 5 (strongly agree)	Study 1: *n =* 202 Study 2: *n =* 536	Study 1: NR Study 2: 33.24	Construct			
To conceptualize a suitable measure for the employee wellbeing construct and validate this tool in Indian workplace settings, especially with reference to Information Technology (IT)/Information Technology Enabled Services (ITES) and Banking, Financial Services & Insurance (BFSI) sectors		NOTE: final questionnaire is not published in Khatri and Gupta (55)		% Female: Study 1: 33% Study 2: 37%				
Kun et al. (56)	An employee wellbeing questionnaire using Seligman's multidimensional PERMA model (Positive emotion, Engagement, positive Relationships, Meaning, and Accomplishment) model	35-item, 6-factor scale	Employees from postgraduate courses at the Budapest University of Technology and Economics	Age: 41.38 (7.81)	Structural^*^	Factors: Negative aspects of work: 0.86 Meaning: 0.80 Positive Relationships: 0.83 Engagement: 0.81 Positive emotions (optimism): 0.79 Accomplishment: 0.73	NE	NE
Hungary/Hungarian			*n =* 397	% Female: 61.40%				
To develop a comprehen-sive measure for assessing workplace wellbeing on the basis of positive psychology concerns and the PERMA model								
MacMillan et al. (57)	I-We Wellbeing Survey	6-item scale	Social workers enrolled in an MSW-level social work research course	Age: NR	Structural^*^	Intrapersonal wellbeing: 0.80 Interpersonal wellbeing: 0.84	NE	NE
USA/English	Six items that capture three internal items (i.e., tense–relaxed, stress–calm, tired–energized) and three external items (i.e., disempowered–empowered, disconnectedness–connectedness, and isolated–in community)							
To examine a measure of wellbeing, empowerment, and connectedness after a group recreational drumming intervention with social workers				% Female: 82.20%				
Parker and Hyett (58)	Work Wellbeing Questionnaire	31-item scale with 4 domains	Refinement of measure study: employees exposed to a wide variety of workplace conditions and not necessarily representative of any one workplace environment	Age	Structural^*^	NE	NE	NE
Australia/English	A brief self-report measure capturing several constructs of wellbeing and positive psychology that might be relevant to the workplace setting	5-point scale: 0, not at all; 1, slightly; 2, moderately; 3, very; and 4, extremely true.	Test-retest study: patients being managed for a depressive condition	NR				
To develop a comprehensive, self-report measure assessing workplace wellbeing			Refinement of measure study: *n =* 150 Test-retest reliability study: *n =* 30 Calibration study: *n =* 1,218	% Female Calibration study: 68.2%				
Porath et al. (59)	10-item thriving at work scale	10-item scale measuring two dimensions of thriving: vitality (5 items) and learning (5 items)	Populations studied: Pilot study: workers in a variety of occupations (e.g. consultant, engineer, administrator)Study 1: Sample 1: undergraduate students enrolled in a senior-level business course at a large Western university in the USA Sample 2: Young Professionals and their supervisors Study 2: Three samples: physical facilities sample, composed of employees primarily performing manual labor; multicompany, professionals sampled from six different organizations across a variety of industries; executive Master of Business	Age Pilot study: 27.8 (NR) Study 1 Sample 1: 21 (2.28) Study 1 Sample 2: 28.3 (2.87) Study 2 Sample 1: 43 (9.88) Study 2 Sample 2: NR Study 2 Sample 3: 32.72 (5.72) Study 3: % Female	Structural	Study 2 Plant Facilities sample: 0.93 Multicompany sample: 0.90 MBA degree sample: 0.94	NE	NE
USA/English	A measure of the construct of thriving at work	7-point scale (1 = strongly disagree to 7 = strongly agree)	Administration degree (EMBAs), consisting of managers completing the final course of their program	Study 1: Sample 1: 37% Sample 2: 34%	Construct			
To develop and validate a measure of the construct of thriving at work			*N* Pilot study *n =* 30 Study 1: Sample 1 *n =* 175 Sample 2 *n =* 410 Study 2 Sample 1: *n =* 616 Sample 2: *n =* 335 Sample 3: *n =* 136 Study 3 *n =* 335	Study 2: Physical facilities sample: 25% Multicompany sample: 31% Executive Master of Business Administration degree sample: 34%				
Pradhan and Hati (60)	Employee Wellbeing Scale	31-item scale measuring four dimensions of employee wellbeing (WB)	IT and HR professionals	Age: NR	Structural	Psychological WB: 0.95 Social WB: 0.72 Subjective WB: 0.90 Workplace WB: 0.95	Pearson's correlation between time 1 and 2 (40 days): 0.733, *p* < 0.01	NE
India	A multidimensional scale of employee wellbeing		Study 1: *n =* 117 Study 2: *n =* 316 Study 3: *n =* 123	% Female Study 1: 41.89% Study 2: 40.16% Study 3: 47.96%	Construct			
To expand the understanding on the structural dimensions of employee wellbeing and develop a comprehensive scale to objectively measure the phenomena of employee wellbeing								
Watanabe et al. (61)	The University of Tokyo Occupational Mental Health (TOMH) wellbeing 24 scale	24-item, 8-factor scale	Workers who lived in all prefectures in Japan	Age Survey 1: 45.09 (13.7)	Structural	Subscales: Role-oriented future prospects: 0.82 Autonomy: 0.76 Role-oriented positive perception: 0.86 Personal Growth and Development: 0.80 Negative Schema: 0.74 Occupational Self Esteem: 0.85 Relationship: 0.77 Meaningful Work: 0.83	ICC (2-week interval): Role-oriented future prospects: 0.738 Autonomy: 0.749 Role-oriented positive perception: 0.731 Personal Growth and Development: 0.746 Negative Schema: 0.671 Occupational Self Esteem: 0.626 Relationship: 0.695 Meaningful Work: 0.781	SEM ranged from 0.486 to 0.661
Japan/Japanese	A tool for eudemonic wellbeing at work, as an independent concept from general eudemonic wellbeing	7-point Likert scale (0 = strongly disagree, 6 = strongly agree)	Study 1: *n =* 1,030 Study 2: *n =* 730	Survey 1: follow-up: 44.39 (10) Survey 2: 45.14 (14.1)	Construct			SDC ranged from 1.348 to 1.831, indicating that an approximate 1.5-point change of scores implies meaningful change of the concepts
To develop a new tool to measure eudemonic wellbeing at work				% Female Survey 1: 50.5% Survey 1 follow-up: 45.1% Survey 2: 50.4%				
Zheng et al. (62)	Employee wellbeing (EWB) scale	18-item EWB scale with three subscales: Life wellbeing Work wellbeing Psychological wellbeing	Qualitative study: employees from one coal mine company in China	Age	Structural	EWB instrument: 0.93	Pearson's correlation between time 1 and 2 (1 month apart): 0.73 *p* < 0.01	NE
China and USA/Chinese and English	A measure of employee wellbeing with three dimensions: life wellbeing, workplace wellbeing, and psychological wellbeing	A Likert 7-point scale (1 = strongly disagree, 7 = strongly agree)	Study 1: managers Study 2: employees from an airline company in China Study 3: employees, from four firms in China from industries, including energy (16%), consulting (13.8%), construction (5.2%), and restaurant services (65%) Study 4: employees from one high-tech company in China Study 5: employees from one equipment manufacturing company in China Study 6: employees from one outdoor sport clothing manufacturing company in China Study 7: employees from United States	Qualitative study: 32.3 Study 1: 34.4 Study 2: 32.4 Study 3: 29.6 Study 4: 28.45 Study 5: 24.4 Study 6: 29.7 Study 7: 40.6	Construct	Subscales: Workplace wellbeing 0.93 Life wellbeing 0.92 Psychological wellbeing 0.88		
To explore the connotations and structural dimensions of employee wellbeing and develop a relevant measure using Chinese samples			Qualitative study: *n =* 310 Study 1: *n =* 400 Study 2: *n =* 295 Study 3: *n =* 424 Study 4: *n =* 217 Study 5: *n =* 290 Study 6: *n =* 277 Study 7: *n =* 250	% Female Qualitative study: 19.2% Study 1: 27.8 Study 2: 48.1 Study 3: 48.3 Study 4: 47.4 Study 5: 36.3 Study 6: 46.6 Study 7: 39.2	Cross cultural/measurement invariance			
Zhou and Parmanto (63)	Pitt Wellness Scale	44-item, 7-factor scale	Current University of Pittsburgh students, staff, and faculty	Age	Structural	Pitt Wellness Scale Instrument: 0.93	NE	NE
USA/English	A comprehensive well-being scale for people in a university environment, including students, faculty, and staff	A scale from 1 (strongly agree) to 7 (strongly disagree)	Study 1: 41.6 (13.4) Study 2: 44 (12.99) Study 3: 43.7 (13.54)	Physical domain: 0.71			
To design and validate a comprehensive well-being scale for people in a university environment, including students, faculty, and staff	Study 1: Staff: *n* = 378 Faculty: *n* = 68 Students: *n* = 66 Study 2: Staff: *n* = 127 Faculty: *n* = 28 Students: *n* = 7 Study 3: Staff: *n* = 370 Faculty: *n* = 113 Students: *n* = 62	% Female Study 1: 78.2% Study 2: 79% Study 3: 75.3%	Mental domain: 0.86 Social domain: 0.78 Financial domain: 0.86 Spiritual domain: 0.89 Occupational domain: 0.84 Intellectual domain: 0.83				

Methodology abbreviations: ICC, Intraclass correlation coefficient; NR, not reported; NE, not evaluated; SDC, smallest detectable change; SEM, standard error of measurement.

* Structural validity assessed only through exploratory factor analysis.

# Studies are reported on these measurement properties but not conducted in the population of interest for the present review.

Other abbreviations: BFSI, Banking, Financial Services & Insurance; EWB, employee wellbeing; EMBA, executive Master of Business Administration degree; IPWBW, index of psychological wellbeing at work; IT, Information Technology; ITES, Information Technology Enabled Services; MSW, master of social work; PVM, proactive vitality management; PWBW, psychological wellbeing at work; TOMH, Tokyo Occupational Mental Health; WB, wellbeing; WRWB, work-related wellbeing.

Of the 18 articles reporting the development of new instruments (46–63), four did so with employees in the United States, two with employees in Australia, and two with employees in India. Eight studies developed instruments with populations in China, Japan, Hungary, the UK, the Netherlands, Sweden, Taiwan, and Canada. Two studies did not report specific country contexts for the participants: Anderson et al. (46) did not report a specific country context and developed their scale with an online survey panel of “employees” who were fluent in English, and Butler and Kern (47) studied a sample of employees from an online company based at several global offices. All studies included male and female participants, and, as expected given the focus on workers, the mean age of participants in studies that reported this parameter tended to be between early thirties to mid-forties.

Eight of these studies developed instruments with relatively heterogeneous samples of workers from a range of industries and across the country of interest (49–51, 55, 58, 60–62). Eight studies developed instruments in well-defined populations in well-defined settings, such as nurses within a specific medical center (48); staff at a university (56, 63); staff at a school or within a specific school system (53, 54); social workers undertaking a specific course (57); staff working in a library service in southern England (52); and employees of one specific online company with a global presence (47). Porath and Hyett (59) report a series of different studies for different measurement properties, with different samples ranging from factory workers to executives.

### 3.2. Assessment of methodological quality

The methodological quality of studies that develop new wellbeing instruments and of the measurement properties of the instruments are summarized in Table 2.

**Table 2:** Methodological quality of *studies* that develop new instruments and of the measurement properties of the *instruments*.

**Instrument [references]**	**Structural validity**		**Internal consistency**		**Cross cultural validity/measurement invariance**		**Reliability**		**Measurement error**		**Criterion validity**		**Construct validity**		**Responsiveness**		**Interpretability**
	**Study**	**Instrument**	**Study**	**Instrument**	**Study**	**Instrument**	**Study**	**Instrument**	**Study**	**Instrument**	**Study**	**Instrument**	**Study**	**Instrument**	**Study**	**Instrument**	**Instruments only**
Personal Growth and Development Scale (PGDS) (46)	Very Good	+	Very Good	+	Very Good	+	N/E	N/E	N/E	N/E	N/E	N/E	Very Good	+	N/E	N/E	Y
PERMA-Profiler (47)	N/E^#^	N/E^#^	Very Good	^*^	N/E	N/E	N/E	N/E	N/E	N/E	N/E	N/E	N/E	N/E	N/E	N/E	Y
Nursing Health and Job Satisfaction (NHJS) scale (48)	Very Good	+	Very Good	+	N/E	N/E	N/E	N/E	N/E	N/E	N/E	N/E	N/E	N/E	N/E	N/E	N
Index of Psychological Wellbeing at Work (IPWBW) (49)	Doubtful^¶^	+	Very Good	+	N/E	N/E	N/E	N/E	N/E	N/E	N/E	N/E	Very Good	+	N/E	N/E	Y
Proactive vitality management (PVM) scale (50)	Very Good	+	Very Good	+	N/E	N/E	N/E	N/E	N/E	N/E	N/E	N/E	Very Good	+	N/E	N/E	Y
Work-related wellbeing (WRWB) index (51)	Doubtful^¶^	+	Very Good	+	N/E	N/E	N/E	N/E	N/E	N/E	N/E	N/E	Inadequate	+	N/E	N/E	N
Work and Wellbeing Assessment for Libraries (52)	Inadequate^**^	N/E	Very Good	^*^	N/E	N/E	N/E	N/E	N/E	N/E	N/E	N/E	N/E	N/E	N/E	N/E	Y
Occupational Social Wellbeing Inventory (OSWI) (53)	Adequate	N/E^†^	Very Good	^*^	N/E	N/E	N/E	N/E	N/E	N/E	N/E	N/E	Very Good	+	N/E	N/E	Y
A scale based on Seligman's (41) multidimensional PERMA (54)	Inadequate	+	Very Good	+	N/E	N/E	N/E	N/E	N/E	N/E	N/E	N/E	N/E	N/E	N/E	N/E	Y
Employee wellbeing scale (55)	Very Good	?	Very Good	?	N/E	N/E	N/E	N/E	N/E	N/E	N/E	N/E	Inadequate	+	N/E	N/E	N
An employee wellbeing questionnaire using multidimensional PERMA model (56)	Adequate	N/E^†^	Very Good	^*^	N/E	N/E	N/E	N/E	N/E	N/E	N/E	N/E	N/E	N/E	N/E	N/E	N
I-We Wellbeing Survey (57)	Adequate	N/E^†^	Very Good	^*^	N/E	N/E	N/E	N/E	N/E	N/E	N/E	N/E	N/E	N/E	N/E	N/E	Y
Work Wellbeing Questionnaire (58)	Inadequate	N/E^†^	Inadequate	N/E	N/E	N/E	N/E	N/E	N/E	N/E	N/E	N/E	N/E	N/E	N/E	N/E	Y
10-item scale measuring two dimensions of thriving (59)	Very Good	+	Very Good	+	N/E	N/E	N/E	N/E	N/E	N/E	N/E	N/E	Very Good	+	N/E	N/E	Y
Employee Wellbeing Scale (60)	Doubtful^¶^	+	Very Good	+	N/E	N/E	Doubtful	?	N/E	N/E	N/E	N/E	N/E	N/E	N/E	N/E	Y
The University of Tokyo Occupational Mental Health [TOMH] wellbeing 24 scale (61)	Very Good	+	Very Good	+	N/E	N/E	Adequate	+	Adequate	+	N/E	N/E	Very Good	+	N/E	N/E	Y
Employee wellbeing (EWB) scale (62)	Very Good	+	Very Good	+	Inadequate	+	Doubtful	?	N/E	N/E	N/E	N/E	Very Good	+	N/E	N/E	Y
Pitt Wellness Scale (63)	Doubtful^¶^	+	Very Good	+	N/E	N/E	N/E	N/E	N/E	N/E	N/E	N/E	N/E	N/E	N/E	N/E	Y

Quality criteria for studies: “Very Good,” “Adequate,” “Doubtful” or “Inadequate” quality according to COSMIN Risk of Bias Assessment; Quality criteria for measurement properties of instruments: Sufficient (+); indeterminate (?); insufficient (–) according to Mokkink et al. (20).

N/E, means the measurement property was not explored in the included study; Interpretability: Y indicates that “Yes” at least some descriptive statistics are reported for the instrument, N indicates that “No” descriptive statistics are reported.

# Study reported measurement properties not evaluated here because these studies were conducted on samples that were not primarily workers.

* Indicates that internal consistency for scale was reported, but in accordance with the COSMIN scoring criteria, cannot be interpreted due to absence of evidence for structural validity with confirmatory factor analysis (either because only exploratory factor analysis was carried out, or because no factor analysis was carried out).

** Indicates no factor analysis was carried out.

† Structural validity was explored but measurement property cannot be appraised because only exploratory not confirmatory factor analysis was carried out.

¶ Indicates “Doubtful” rating because exploratory and confirmatory factor analysis were performed on same sample but otherwise all criteria were met for “Very Good” were met and so evaluated the measurement property of structural validity.

Each of the 18 articles reported the development of a single new instrument, and thus there were 18 new instruments identified. Within the articles the number of studies investigating the measurement properties of these new instruments in a sample of workers ranged from one (47) to five (61, 62). Butler and Kearn (47) report studies investigating other measurement properties, but these studies were carried out in different samples that were not exclusively comprised of workers. Across the 18 articles, the methodological quality of studies of measurement properties ranged from “Very Good” to “Inadequate.” The most frequently explored measurement properties for development of a new instrument were structural validity and internal consistency; in contrast, some measurement properties in the COSMIN taxonomy (18) were studied infrequently (e.g., responsiveness, measurement invariance) or not at all (e.g. criterion validity).

Of the 18 articles, only four explicitly stated that a pilot test was conducted with the target population to check item comprehensiveness and comprehensibility (52, 59, 60, 63). Conducting a pilot test in the target population (workers or employees) is one of the standards for rating an instrument development study as “Very Good” as opposed to “Inadequate.” These four studies were then assessed according to the extent to which they met the remaining standards for PROM development methodological quality (19, 21, 24). Juniper et al. (52) did not specify the sample size used for the pilot study (i.e., stating only that “*The questionnaire was pre-tested with a number of library staff to ensure content and instructions were clear*.”; p. 110), and so overall the methodological quality of this PROM development study is rates as “Doubtful.” Porath et al.'s (59) pilot study for comprehensiveness/clarity employed an adequate methodology but a sample size of only 30, so instrument development was rated as “Doubtful.” Pradhan and Hati (60) also reported both eliciting concepts through interviews and testing items for clarity and comprehensiveness in an adequate sample from the target population, and so was rated as “Very Good.” Zhou and Parmanto (63) development of the Pitt Wellness Scale was rated as “Very Good,” given its detailed methodology and description of the pilot testing process, and the samples used in these processes.

Three of the remaining 14 articles (49, 55, 62) did involve the target population in concept elicitation through interviews. However, these researchers developed items based on these concepts and proceeded to administer the instrument and explore measurement properties without testing the clarity or comprehensiveness of the individual items with the target population.

The 11 articles left did not refer to a target population involvement at any point during either concept elicitation or pilot testing items for comprehensibility and comprehensiveness. Item generation was informed exclusively by the researchers', and in some cases their colleagues', expertise and familiarity with the literature (46, 48, 50, 53, 56, 58, 61), or based on items from existing “wellbeing” instruments. However, pilot testing the items for comprehensiveness and comprehensibility in the new context did not occur (47, 51, 54, 57).

#### 3.2.1. Structural validity

Of the 18 studies that developed new instruments, one [Butler and Kern (47)] was excluded from our evaluation of structural validity because a mixed sample of employed and unemployed participants were used for the study of this specific measurement property. Of the 17 included studies that were evaluated for this measurement property, three were rated as “Inadequate,” four as “Doubtful,” three as “Adequate,” and seven as “Very Good.” The three studies evaluated as “Inadequate” included Juniper et al. (52), who did not use factor analysis as a method; and Kern et al. (54) and Parker and Hyett (58), whose sample size was less than five times the number of items. A common reason for the “Doubtful” ratings included failure to use separate samples for the exploratory and confirmatory factor analysis stages (49, 51, 60, 63). Three were rated as “Adequate” because they performed only exploratory but not confirmatory factor analysis (53, 56, 57). Seven studies of structural validity were rated as “Very Good” (46, 48, 50, 55, 59, 61, 62).

Structural validity measurement properties were evaluated for 14 of the included instruments. The other four studies included two studies in which only exploratory factor analysis was carried out, one study that employed a different method of structural validity assessment, and one study that established this measurement property in a mixed sample including, but not exclusively composed of, workers. Of the remaining 11 studies that conducted factor analysis, all but one instrument met the criteria for a rating of “Sufficient” for the measurement property of structural validity; for the Employee Wellbeing Scale (55), the structural validity measurement property was rated as “Indeterminate” because the factor analysis model fit parameters required according to the COSMIN guidelines were not reported.

#### 3.2.2. Internal consistency

For all but one (58) of the new wellbeing instruments, studies were carried out to determine internal consistency. All were methodologically rated as “Very Good.” Evaluation of the measurement property of internal consistency requires at least low evidence for sufficient structural validity, and therefore only 11 instruments were evaluated for the internal consistency measurement property; for all of these, internal consistency was rated as “Sufficient.” Although five additional studies report data for the internal consistency of the instrument, this measurement property was not evaluated in the present review because there was not at least low level of evidence for structural validity based on the methods used (52, 53, 56, 57) or because structural validity assessment had been performed in a non-worker population (47).

#### 3.2.3. Construct validity

When evaluating studies of construct validity we found researchers used a wide range of methods to evaluate to construct validity, for example, convergent validity (59, 61, 62), nomological validity (50), and concomitant validity (49). Some studies investigation of criterion validity was not in accordance with the COSMIN definition of the term, but was better aligned with a study on construct validity. These studies evaluation of criterion validity were considered to be construct validity evidence when considering the COSMIN criteria for construct validity.

Eight of the included articles that developed new instruments conducted studies of what we deemed investigation of construct validity (46, 49–51, 55, 59, 61, 62). Six met all of the COSMIN methodological standards for a rating of “Very Good” (46, 49, 50, 59, 61, 62). Two were rated as methodologically “Inadequate” because measurement properties of the comparator or related measurement instrument(s) were not adequately reported (51, 55). For all eight instruments for which construct validity was assessed, the measurement property of construct validity was rated as “Sufficient,” though given the risk of bias in the construct validity studies of the instruments of Eaton et al. (51) and Khatri and Gupta (55), further exploration is warranted.

#### 3.2.4. Other measurement properties

Several measurement properties were explored infrequently, including (test-retest) reliability, measurement error, measurement invariance, and responsiveness. Watanabe et al. (61) conducted a study of test-retest reliability and report the intraclass correlation coefficient results; however, given the absence of comments regarding the stability of respondents between timepoints and the similarity of the testing conditions, this study was rated as “Adequate.” Six out of eight subscales in Watanabe et al.'s (61) instrument had ICCs >0.7, so overall the measurement property of test-retest reliability was rated as “Sufficient.” Both Pradhan and Hati (60) and Zheng et al. (62) conducted test-retest reliability studies that were appraised as being of “Doubtful” quality, because the ICC was not determined, and rather the Pearson's correlation coefficient was reported without providing evidence that no systematic change had occurred between each timepoint of the tests. Given the absence of a reported ICC, the measurement property of test-retest reliability for the instruments of both Pradhan and Hati (60) and Zheng et al. (62) are rated as “Indeterminate.” None of these studies commented specifically on the stability of the participants between repeated measurements. Zheng et al. (62) investigated measurement invariance in their development of the instrument however, this was rated as “Inadequate” quality because it did not meet the criteria of ensuring that samples were similar in all ways except for the cultural context. Watanabe et al. (61) investigated measurement error of the Japanese version of the PERMA Profiler; this was rated as “Adequate,” lacking detail regarding the stability of the employees between the two time points. The measurement error of this scale was rated as “Indeterminate” because the MIC was not reported. Responsiveness was not determined for any instrument. Evidence of responsiveness is a measurement property lacking from the series of wellbeing instruments developed in the 2010–2020 decade.

#### 3.2.5. Interpretability

Terwee et al. (33) specify that adequate instrument interpretability requires information and means and standard deviations in multiple groups, as well as the minimally important change (MIC). None of the included studies reported MIC so, technically, none of the instruments should be rated as favorable. However, for the purposes of this review, interpretability was rated as either positive or negative, with a positive rating being applied if at least means and standard deviations were reported. Four studies in which new instruments were developed did not report any descriptive statistics for the scores produced from the instrument being developed (48, 51, 55, 56). All other authors report some descriptive statistics (at least means and standard deviations) for the scale being developed, in some cases for individual items and/or factors within the scale, and in some cases for individual subgroups within the broader sample, for example, for males and females separately (53) or for different groups depending on duration of work (52).

### 3.3. Characteristics of articles reporting psychometric validation of previously developed instruments

Eleven articles reported validation of wellbeing instruments originally developed before 2010 and/or were previously developed or validated in a different population or context (64–74). These 11 articles reporting psychometric evaluations of previously developed wellbeing instruments are summarized in Table 3.

**Table 3:** Characteristics of articles reporting psychometric validation of previously developed instruments.

**References**	**Instrument**	**Version**	**Study**	**Demographics**	**Validity**	**Internal**	**Test-retest**	**Measurement**
**Country/language of**			**population**	**Age: mean (SD)**	**Validity**	**consistency**		**error**
**study**			***N***			**(α)**		
**Aim of study**				**% Female**				
Demo and Paschoal (64)	Wellbeing at Work Scale (WBWS)	29-item, 3-factor scale	Employees from a wide range of industries in the United States	Age: N/R	Structural	Factors: Positive Affect: 0.92 Negative Affect: 0.94 Fulfillment: 0.92	N/E	N/E
USA/English	Originally developed by Paschoal and Tamayo (76)		Study 1: *n =* 409 Study 2: *n =* 400	% Female Study 1: 33% Study 2: 42%	Construct			
Aim: to look for evidence of validity in the US regarding the wellbeing at work scale, which was first validated in Brazil to measure employee wellbeing perceptions								
Gurková et al. (65)	Personal Wellbeing Index (PWI)	Slovak and Czech versions	Hospital staff nurses from 12 hospitals in the Czech and Slovak Republics	Age: 39.8 (10.06)	Structural	Czech: 0.85	N/E	N/E
Slovakia and Czech Republic/Slovak and Czech	Originally designed by Cummins et al. (77)		*n =* 1,043	% Female: 98.40%	Construct	Slovak: 0.86		
Aim: Investigate the psychometric properties of the Slovak and Czech versions of PWI in population of nurses in both countries								
Laguna et al. (75)	Job-related affective wellbeing scale (12-item)	Spanish, Polish and Dutch versions	Employees from three countries (Netherlands, Poland, Spain) from small- and medium-sized enterprises	Age Spanish: 40.44 (9.31) Dutch: 44.07 (11.28) Polish: 40.36 (11.19)	Structural	Spanish: 0.65 to 0.84 Dutch: 0.65 to 0.83 Poland: 0.78 to 0.90	N/E	N/E
Netherlands, Poland, Spain/Dutch, Polish, Spanish	Originally designed by Warr (78)		Spanish *n =* 207 Dutch *n =* 254 Polish *n =* 346	% Female Spanish: 37.7% Dutch: 34.6% Polish: 47%	Construct			
Aim: Test the measurement invariance of the instrument across cultures					Cross-cultural validity\measurement invariance			
Lorente et al. (67)	Spanish Orientation to Happiness Scale	Spanish	Spanish workers	Age	Structural	Composite reliability: Hedonic factor: 0.76 Eudemonic factor: 0.73	N/E	N/E
Spanish	Originally designed by Peterson et al. (79)			N/R	Construct			
Aim: Adapt and validate the Spanish Orientations to Happiness Scale				% Female: 54.5				
Love et al. (68)	WHO-5 and WHO-10	Swedish versions	Three cohorts of Swedes: Randomized general population cohort (*n =* 4,027) Employees sick-listed reported by the employer (*n =* 3,310) Self-certified sick-listed individuals (*n =* 498)	Age Randomized general population cohort: 42 (13.1) Employees sick-listed reported by the employer: 47 (11.8) Self-certified sick-listed individuals: 41 (11)	Structural	WHO-10: Randomized general population cohort: 0.92 Employees sick-listed reported by the employer: 0.92 Self-certified sick-listed individuals: 0.95	N/E	N/E
Sweden/Swedish								
Aim: validate the Swedish translation of the WHO (Ten) and WHO (Five) Wellbeing Questionnaires				% Female Randomized general population cohort: 55% Employees sick-listed reported by the employer: 66.3% Self-certified sick-listed individuals: 65%		WHO-5: Randomized general population cohort:0.83 Employees sick-listed reported by the employer:0.82 Self-certified sick-listed individuals: 0.88		
Mielniczuk and Łaguna (69)	Job-related affective wellbeing Scale	Polish version	Polish employees from various professions	Age: 32.81 (8.8)	Structural	Anxiety: 0.88 Comfort: 0.87 Depression: 0.91 Enthusiasm: 0.93	Pearson correlations: 0.76 for enthusiasm 0.72 for comfort 0.68 for depression 0.65 for anxiety	N/E
Poland/Polish					Construct			
Aim: to test the factorial structure of job-related affect in a Polish sample	Originally designed by Warr (78)			% Female: 65%				
Rautenbach and Rothmann (70)	Flourishing-at-Work Scale Short Form		A stratified random sample of employees of an alcoholic beverage company	Age: N/R	Structural	Emotional WB: 0.77 Psychological WB: 0.89 Social WB: 0.89	N/E	N/E
South Africa	Originally designed by Rautenbach (80)		*n =* 779	% Female: 40.40%				
Aim: validate the Flourishing-at-Work Scale Short Form (FWS-SF) in a South African fast-moving consumable goods industry								
Sandilya and Shahnawaz (71)	The Index for Psychological Wellbeing at Work (IPWBW)		Employees from automobile and automotive parts manufacturers	Age: 33.74	Structural	Instrument: 0.83	N/E	N/E
India/not reported	Designed by Dagenais-Desmarais and Savoie (49)		*n =* 387	% Female: 19%		Subscales: Interpersonal Fit at Work: 0.76 Thriving at work: 0.97 Feeling of Competency at work: 0.79 Perceived Recognition at work: 0.82 Desire for Involvement at work: 0.73		
Aim: to validate an existing tool for the Indian working population								
Senol-Durak and Durak (72)	The Flourishing Scale	Turkish translations	Turkish employed adults.	Age: 34.79 (9.32)	Structural	The Flourishing Scale: 0.89	N/E	N/E
Turkey/Turkish	Originally developed by Diener et al. (81)		*n =* 180		Construct			
Aim: assess the psychometric distinctive features of The Flourishing Scale				% Female: 47.80%	Cross-cultural validity\measurement invariance			
Watanabe et al. (73)	The Workplace PERMA-Profiler	Japanese version	Workers registered as respondents of an Internet survey company	Age Baseline: 44.9 (13.6) Follow-up: 45.8 (13)	Structural	Positive emotion: 0.92	ICCs (1-month interval between T1 and T2): Positive emotion: 0.86 Engagement: 0.83 Relationships: 0.83 Meaning: 0.77 Accomplishment: 0.77	Smallest detectable change: Positive emotion: 2.49 Engagement: 2.42 Relationships: 2.27 Meaning: 2.56 Accomplishment: 2.56
Japan/Japanese	Originally designed by Kern (82)		Baseline *N* = 310		Construct	Engagement: 0.85		
Aim: to investigate the validity of the Japanese version of the Workplace PERMA-Profiler	11-point Likert-type scale (ranging from 0 to 10)		Follow-up: *N* = 86	% Female Baseline: 44.9% Follow-up: 45.8%		Relationships: 0.75		
Weziak-Białowolska et al. (74)	Flourish Index and Secure Flourish Index		Employees of two US Fortune 500 companies	Age: 42 (12.4)	Structural	Flourishing Index: 0.89	N/E	N/E
English/USA	Originally developed by VanderWeele (83) and VanderWeele et al. (84)		*N* = 5,565	% Female: Company 1: 44.5% Company 2: 30.4%%	Construct	Secure Flourishing Index: 0.86		
Aim: to validate the psychometric properties of the Flourish Index (FI) and Secure Flourish Index (SFI) in the workplace setting								

N/E, not explored; N/R, not reported.

One of these articles validated in a US population of workers a wellbeing instrument previously developed in Brazil (64). Several of the included articles undertook validations in new populations of workers in countries/languages that differed from the English/American populations in which the instrument had previously been developed and validated (65, 67–73, 75). Another sought to validate a previously developed instrument specifically in a population of workers (74). None of the included articles assessed content validity, criterion validity (according to the specific definition of criterion validity in the COSMIN guidelines) or responsiveness of the instruments.

### 3.4. Methodological quality of *studies* and appraisal of measurement properties of psychometrically validated *instruments*

The quality appraisal of studies that psychometrically validate previously developed instruments and appraisal of the measurement properties of the instruments are summarized in Table 4.

**Table 4:** Methodological quality of *studies* that psychometrically validate previously developed instruments and measurement properties of the *instruments*.

**Instrument [references]**	**Structural validity**		**Internal consistency**		**Cross cultural validity/ measurement invariance**		**Reliability**		**Measurement error**		**Criterion validity**		**Construct validity**		**Responsiveness**		**Interpretability**
	**Study**	**Instrument**	**Study**	**Instrument**	**Study**	**Instrument**	**Study**	**Instrument**	**Study**	**Instrument**	**Study**	**Instrument**	**Study**	**Instrument**	**Study**	**Instrument**	**Instruments only**
Wellbeing at Work Scale (WBWS), Demo and Paschoal (64)	Very Good	+	Very Good	+	N/E	N/E	N/E	N/E	N/E	N/E	N/E	N/E	Very good	+	N/E	N/E	N
Personal Wellbeing Index (PWI), Gurková, DŽuka (65)	Adequate	N/E^‡^	Very Good	^§^	N/E	N/E	N/E	N/E	N/E	N/E	N/E	N/E	Inadequate	+	N/E	N/E	Y
Job-related Affective Wellbeing Scale, Laguna et al. (75)	Very Good	+	Very Good	+	Very Good	+	N/E	N/E	N/E	N/E	N/E	N/E	N/E	N/E	N/E	N/E	Y
Spanish Orientation to Happiness Scale, Lorente, Tordera (67)	Very Good	+	Very Good	+	N/E	N/E	N/E	N/E	N/E	N/E	N/E	N/E	Very good	+	N/E	N/E	Y
WHO-5 and WHO-10, Love, Andersson (68)	Adequate	N/E^‡^	Very Good	^§^	N/E	N/E	N/E	N/E	N/E	N/E	N/E	N/E	Inadequate	+	N/E	N/E	Y
Job-related Affective Wellbeing Scale, Mielniczuk and Łaguna (69)	Very Good	+	Very Good	+	N/E	N/E	Doubtful	?	N/E	N/E	N/E	N/E	Very good	+	N/E	N/E	Y
Flourishing-at-Work Scale Short Form, Rautenbach and Rothmann (70)	Very Good	+	Very Good	+	N/E	N/E	N/E	N/E	N/E	N/E	N/E	N/E	N/E	N/E	N/E	N/E	Y
The Index for Psychological Wellbeing at Work (IPWBW), Sandilya and Shahnawaz (71)	Very Good	+	Very Good	+	N/E	N/E	N/E	N/E	N/E	N/E	N/E	N/E	N/E	N/E	N/E	N/E	N
The Flourishing Scale, Senol-Durak and Durak (72)	Very Good	+	Very Good	+	Very Good	+	N/E	N/E	N/E	N/E	N/E	N/E	Very good	+	N/E	N/E	Y
The Workplace PERMA-Profiler, Watanabe et al. (73)	Very Good	+	Very Good	+	N/E	N/E	Adequate	+	Adequate	?	N/E	N/E	Very Good	+	N/E	N/E	Y
Flourish Index and Secure Flourish Index, Weziak-Białowolska, Białowolski (74)	Very Good	+	Very Good	+	N/E	N/E	N/E	N/E	N/E	N/E	N/E	N/E	Inadequate	+	N/E	N/E	Y

Assessment criteria for methodological quality of studies: “Very Good,” “Adequate,” “Doubtful,” or “Inadequate” quality according to COSMIN Risk of Bias Assessment; Quality criteria for appraisal of of measurement properties of instruments: Sufficient (+), indeterminate (?), insufficient (–) according to Terwee et al. (23).

CFA, confirmatory factor analysis; EFA, exploratory factor analysis; N/E, not explored means the measurement property was not explored in the included study.

‡ Indicates that EFA (not CFA) was carried out.

§ Indicates that internal consistency for scale was reported, but in accordance with the COSMIN scoring criteria, cannot be interpreted due to absence of evidence for structural validity with confirmatory factor analysis; Y indicates that “Yes” at least some descriptive statistics are reported for the instrument; N indicates that “No” descriptive statistics are reported for the instrument.

Within these 11 validation articles, the number of measurement properties studied for any one instrument ranged from three to four. Structural validity and internal consistency were the most frequently studied properties; measurement error and measurement invariance were infrequently studied; and content validity, criterion validity, reliability and responsiveness were never studied.

#### 3.4.1. Structural validity

Structural validity was investigated in all 11 studies that psychometrically validate previously developed instruments. The methodological quality of these studies was rated as “Very Good” for all except for two rated as “Adequate” (65, 68). The reason for the ratings of “Adequate” was that exploratory, but not confirmatory, factor analysis was carried out. Of the nine instruments for which structural validity studies were carried out with confirmatory factor analyzes, the measurement property of structural validity was rated as “Sufficient” in all (64, 67, 69–75).

#### 3.4.2. Internal consistency

The methodological quality of internal consistency was rated as “Very Good” for all 11 studies (64, 65, 67–75). This measurement property was rated as “Sufficient” for all instruments except for the two for which this measurement property could not be appraised because there was insufficient evidence for structural validity given that it had been assessed with only exploratory but not confirmatory factor analysis (65, 68).

#### 3.4.3. Measurement invariance/cross-cultural validity

Two studies evaluated measurement invariance/cross-cultural validity of previously developed wellbeing instruments; both were methodologically rated as “Very Good.” Laguna et al. (75) validated the Job-Related Affective Wellbeing scale in samples of workers in the Netherlands, Poland and Spain and demonstrated measurement invariance of the instrument across these country contexts. In this study, the property of measurement invariance was rated as “Sufficient.” Senol-Durak and Durak (72) established measurement invariance of the Turkish version of the Flourishing Scale for male and female employees; the measurement property of this scale was rated as “Sufficient.”

#### 3.4.4. Test-retest reliability

Two studies evaluated test-retest reliability of previously developed wellbeing instruments. Mielniczuk and Łaguna (69) conducted a test-retest reliability study of the Job-Related Affective Wellbeing scale in a sample of Polish workers, reporting Pearson's correlation coefficients rather than the COSMIN recommendation of intraclass correlations, so the measurement property of test-retest reliability was “Indeterminate”; furthermore, Mielniczuk and Łaguna (69) did not comment on stability of the respondents in the intervening period, and so the methodological quality was rated as “Doubtful” according to the COSMIN criteria. Watanabe et al. (73) undertook a test-retest reliability study of the Japanese Workplace PERMA-Profiler, and although they did report the reliability in the COSMIN-recommended manner of intraclass correlations and this parameter was of a sufficient value, they did not comment on the stability of the respondents between the two testing sessions, and so overall the methodological quality of the study on this measurement property was rated as “Adequate.”

#### 3.4.5. Hypothesis testing for construct validity

Eight of the 11 studies investigated construct validity, although reported this using a variety of terms besides “construct validity.” Several of the studies failed to adequately report measurement properties (i.e., descriptive statistics, internal consistency) in the study population for the comparator instruments used in the convergent validity assessment, and so were rated as “Inadequate” (65, 68, 74). The measurement property of construct validity for all eight instruments for which it was assessed met the COSMIN criteria for “Sufficient”; however, given the “Inadequate” rating of the methodological quality for three of the studies (65, 68, 74), the measurement property of construct validity for these instruments should be treated with caution.

#### 3.4.6. Measurement error

Only Watanabe et al. (73) undertook a study of measurement error for the Japanese Workplace PERMA-Profiler. The methodological quality of this study was rated as “Very Good”; however, the property of measurement error of the Japanese Workplace PERMA-Profiler is rated as “Inconclusive” because the MIC was not reported.

#### 3.4.7. Interpretability

All but two articles (64, 71) report at least some descriptive statistics (mean and standard deviation) for the validated instruments; in some cases, descriptive statistics for scores were reported for individual items and factors within the overall instrument. Some report descriptive statistics from the instruments for different subgroups, such as males vs. females (68) or for different country contexts (75). None of the articles that validated previously instruments investigated or reported data that would help interpret change score (i.e., the minimal important change, or MIC).

## 4. Discussion

This review had four objectives. First, to systematically identify articles published from 2010 to 2020 reporting the development of instruments to measure workers' wellbeing. Second, to critically appraise the methodological quality of the *studies* reporting the development of workers' wellbeing measures. Third, to critically appraise the psychometric properties of the *measures* developed for workers' wellbeing. Fourth, based on the measures developed between 2010 and 2020, recommend valid and reliable measures of workers' wellbeing.

We screened 8,178 articles, and identified 18 articles reporting development a new instrument to measure workers' wellbeing, and 11 that validated existing measures of wellbeing in workers. Numerous articles were excluded due to measuring constructs other than wellbeing, such as illbeing (e.g. burnout). A number of included instruments had subscales that measured constructs related to wellbeing (e.g., job satisfaction) alongside subscales measuring wellbeing. Notable in our review were the different definitions of wellbeing and consequently the different types of content employed by test developers to represent the construct of wellbeing. Whilst variance in content is a threat to the validity of measures, without an agreed upon definition of wellbeing for workers from the population it concerns (workers themselves), validity will always be attenuated. The newly developed measures group and the previously developed group of measures were appraised using their respective COSMIN quality checklists.

### 4.1. Methodological quality

Overall the psychometric studies were insufficient to establish the validity of the measures, whether developed between 2010 and 2020, or previously developed before 2010 (or in a different context). In both the newly developed measures and previously developed measures groups, few studies reported the prevalence of missing data or how any missing data was handled, potentially introducing bias if data is systematically missing (85). Furthermore, statistics used in the analysis were often not clearly reported, omitting details such as the statistical procedures used, rotational methods, or formulas. This creates difficulty in appraising the quality of evidence. No study completed all eight categories to enable a full risk of bias assessment. Whilst exploratory and/or confirmatory factor analysis was often used, hypotheses for CFA were rarely provided and some studies used small samples. Commonly, test-retest reliability, criterion validity, measurement error, responsiveness, and cross-cultural validity were omitted altogether. These steps scaffold together to ensure that the risk of bias is reduced, and omission of a number of these steps as was the case here, has reduced the quality of the studies.

Internal consistency was assessed in all studies despite having a number of limitations for determining reliability [e.g., see (86)]. All except two studies were appraised as having very good internal consistency. Commonly the measurement properties of responsiveness, criterion validity, or content validity were overlooked, and measurement error was rarely reported. For example, measurement error was studied for only one newly developed instrument (61) and was rated as “Indeterminate” for the instruments studied, because the minimally important change was not reported. The lack of evaluation of responsiveness in workers' wellbeing measures is problematic as it does not enable confidence in the instrument's validity if using to assess the impact of interventions on workers' wellbeing.

Our review highlighted a lack of ongoing validation of existing measures, with few studies completing more than three of the nine methods for establishing methodological quality. No studies of content validity were reported in the 11 articles that established measurement properties in instruments originally developed in a different context. This may reflect an implicit assumption by the researchers that the instrument for which they were establishing measurement properties in a new context/population must have content validity in the new population. Many of the instruments for which measurement properties were reported for new contexts, are in common use (e.g., the Job-Related Affective Wellbeing Scale, the WHO-5, and the Warwick Edinburgh Mental Wellbeing Scale). However, it is recommended that studies establish content validity to ensure items retain their validity in a new context (20). As with newly developed instruments, this group of instruments also neglected to assess measurement error, with one validation study of a previously developed instrument reporting measurement error but not minimally important change (73).

### 4.2. Recommendations of valid and reliable measures of workers' wellbeing

We aimed to synthesize evidence from 2010 to 2020 for workers' wellbeing instrument measurement properties in order to recommend valid and reliable measures. No measure achieved the stringent criteria used in the present review for several reasons. First, validation of a new measure generally requires multiple studies and should be conducted in the population where the measure is intended to be used. Second, the studies themselves did not reach the quality standard necessary. Third, the repetition of studies requires time to complete and then publish, resulting in a lag. The overarching reason this study was undertaken was to support researchers in determining the best available measure to use in workers wellbeing research. Consequently, we now make recommendations based on the best available evidence with the caveat of, no measure met the standard set by COSMIN methodology.

Considering the overall evidence for measurement properties for individual instruments, those with the greatest number of positively rated measurement properties amongst newly developed instruments were: (1) Anderson et al.'s (46) Personal Growth and Development Scale (PGDS), for which structural validity, internal consistency, construct validity and measurement invariance were all rated as sufficient; (2) Watanabe et al.'s (61) The University of Tokyo Occupational Mental Health [TOMH] wellbeing 24 scale, for which structural validity, internal consistency, construct validity and reliability were all rated as sufficient, and Item Response Theory (IRT) methods were employed during evaluation; and (3) Zheng et al.'s (62) Employee Wellbeing (EWB) scale, for which structural validity, internal consistency, construct validity were all rated as sufficient. However, none of these newly developed workers' wellbeing instruments met the COSMIN criteria for adequate instrument design.

The Personal Growth and Development Scale (PGDS) (46) was reported to measure perceptions of personal growth and development at work, and was developed based on Ryff's general model and items were developed and refined by subject matter experts. The instrument was tested on employees and students through correlating with constructs of interest, and structural invariance testing was undertaken with scalar invariance found longitudinally within groups, but not between groups. Moderate positive correlations were found between employee responses on the PGDS and Basic Needs Satisfaction, Intrinsic Motivation, Identified Regulation, and Satisfaction with Life. The PGDS is a promising measure that requires ongoing validation in worker samples, as predictive validity was only undertaken in the education version. Anderson et al.'s (46) Personal Growth and Development Scale could be considered for assessing workers' personal growth and development.

Watanabe et al.'s (61) University of Tokyo Occupational Mental Health [TOMH] wellbeing 24 scale was developed in a methodologically sound way that included IRT and Classical Test Theory (CTT) methods. It was developed specifically in workers, and could be considered for applications that aim to specifically assess wellbeing at work, as an independent concept from general eudemonic wellbeing. Watanabe et al. found their measure had overlapping constructs with Ryff's model of wellbeing and Self Determination Theory.

Zheng et al.'s (62) Employee Wellbeing Scale (EWS) was methodologically strong in its development, including items from workers and literature prior to psychometric refinement, strengthening its content validity. The EWS had moderate correlations with related wellbeing constructs and could be considered for assessing dimensions of worker wellbeing such as life wellbeing, workplace wellbeing, and psychological wellbeing. Configural invariance was found between worker samples from China and the United States despite cultural differences, suggesting elements of wellbeing may transcend culture.

Amongst studies of psychometric validation of instruments originally developed before 2010 (or in different context), the best available evidence was for the (1) Flourishing Scale; (2) Workplace PERMA Profiler, (3) Spanish Orientation to Happiness Scale, and (4) the Job Related Affective Wellbeing Scale. As was the case in the newer instruments, validation studies were predominated by CTT methods of evaluation.

### 4.3. Recommendations for future research

A key recommendation based on the findings of this review is that future instrument development studies (1) include the target population throughout the stages of concept elicitation and, subsequently, in pilot testing items for relevance, comprehensiveness and comprehensibility; (2) include samples of an adequate size during the development stage; and (3) describe instrument development methods in adequate detail. Given that most of the measurement properties of worker wellbeing instruments developed between 2010 and 2020 are not reported, there are many opportunities for establishing and validating other measurement properties of recently developed instruments. A consideration for future research is that IRT methods should be used in the development and evaluation of measures. The present research found that studies mainly relied on CTT which has a number of limitations that IRT methods overcome. Although COSMIN does not suggest IRT over CTT, IRT methods such as Rasch analysis are increasingly being used in psychology to increase measurement precision (87). No study reported MIC, the smallest *within-person* change over time above which patients, or, in the context of the current review, employees perceive themselves importantly changed (34). Future studies may explore how this property could be defined, which in turn will enable future research using workers' wellbeing instruments to infer meaningful changes in workers' wellbeing as a result of interventions or changes in circumstances.

### 4.4. Strengths and limitations

The strengths of this review are the use of COSMIN methodology and criteria for assessing studies of measurement properties and the measurement properties of instruments to support rigor and transparency in the review, like resources such as the Cochrane Handbook for Systematic Review and PRISMA guidelines have done for strengthening rigor and transparency in systematic reviews of interventions (as just one example).

The main limitations of this review relate to subjectivity. Despite the use of the COSMIN guidelines, there is still some subjectivity in identifying studies about specific measurement properties, given the diverse names for measurement properties that are used by researchers and that do not align with Mokkink et al.'s (18) taxonomy. Additionally, the use of just three databases and exclusion of studies not reported in the English language contributed to a potential selection bias, particularly associated with studies validating previously developed measures in new languages.

## 5. Conclusion

This review has elucidated the specific measures of workers' wellbeing developed and reported in the decade of 2010 to 2020 and assessed both risk of bias of studies reporting measure development and the quality of measurement properties. This synthesis is an important first step to support future workers' wellbeing researchers to identify and select the most appropriate instruments for effectiveness evaluations. Employing a standardized taxonomy and methodological approach in a globally cohesive and targeted manner will strengthen future scientifically informed developments in workers' wellbeing measurement.

## Data availability statement

The original contributions presented in the study are included in the article/Supplementary material, further inquiries can be directed to the corresponding author.

## Author contributions

Conceptualization: RJ, MS, RS, and JK-M. Data curation: RJ, MS, and HB. Formal analysis and investigation: RJ, MS, HB, and RS. Project administration and resources: RJ. Writing—original draft and writing—review and editing: RJ, HB, MS, RS, and JK-M. All authors contributed to the article and approved the submitted version.
