# Supplementary material for: Wellbeing measures for workers: a systematic review and methodological quality appraisal
Source: Front Public Health. 2023 May 24;11:1053179. doi: 10.3389/fpubh.2023.1053179 (PMC10244676; doi:10.3389/fpubh.2023.1053179)
Supplement: Supplementary file 1 [file Table_1.DOCX]

Supplementary Material

### Supplementary file 1. Search terms executed across databases

#### Health and psychosocial instruments

(((wellbeing or "well being" or well-being) and (employee or employees or worker or workers or staff or personnel) and (instrumentation or methods or "Validation Studies" or "Comparative Study" or "psychometrics" or psychometr* or clinimetr* or clinometr* or "outcome assessment (health care)" or "outcome assessment" or "outcome measure*" or "observer variation" or "Health Status Indicators" or "reproducibility of results" or reproducib* or "discriminant analysis" or reliab* or unreliab* or valid* or "coefficient of variation" or coefficient or homogeneity or homogeneous or "internal consistency" or (cronbach* and (alpha or alphas)) or (cronbach* and (alpha or alphas)) or (item and (correlation* or selection* or reduction*)) or agreement or imprecision or "precise values" or test-retest or ((test and retest) or (reliab* and (test or retest))) or stability or interrater or inter-rater or intrarater or intra-rater or intertester or inter-tester or intratester or intra-tester or interobserver or inter-observer or intraobserver or intra-observer or intertechnician or inter-technician or interexaminer or inter-examiner or intraexaminer or intra-examiner or interassay or inter-assay or intraassay or intra-assay or interindividual or inter-individual or intraindividual or intra-individual or interparticipant or inter-participant or intraparticipant or intra-participant or kappa or kappa's or kappas or repeatab* or ((replicab* or repeated) and (measure or measures or findings or result or results or test or tests)) or generaliza* or generalisa* or concordance or (intraclass and correlation*) or discriminative or "known group" or "factor analysis" or "factor structure" or "factor structures" or dimension* or subscale* or (multitrait and scaling and (analysis or analyses)) or "item discriminant" or "interscale correlation*" or error or errors or "individual variability" or "interval variability" or "rate variability" or (variability and (analysis or values)) or (uncertainty and (measurement or measuring)) or "standard error of measurement" or sensitiv* or responsive* or (limit and detection) or "minimal detectable concentration" or interpretab* or ((minimal or minimally or clinical or clinically) and (important or significant or detectable) and (change or difference)) or (small* and (real or detectable) and (change or difference)) or "meaningful change" or "ceiling effect" or "floor effect" or "item response model" or IRT or Rasch or "differential item functioning" or DIF or "computer adaptive testing" or "item bank" or "cross-cultural equivalence")) not (addresses or biography or "case reports" or comment or directory or editorial or festschrift or interview or lectures or legal cases or legislation or letter or news or newspaper article or patient education handout or popular works or congresses or consensus development conference or practice guideline)).ab.

#### APA PsycInfo

(((wellbeing or "well being" or well-being) and (employee or employees or worker or workers or staff or personnel) and (instrumentation or methods or "Validation Studies" or "Comparative Study" or "psychometrics" or psychometr* or clinimetr* or clinometr* or "outcome assessment (health care)" or "outcome assessment" or "outcome measure*" or "observer variation" or "Health Status Indicators" or "reproducibility of results" or reproducib* or "discriminant analysis" or reliab* or unreliab* or valid* or "coefficient of variation" or coefficient or homogeneity or homogeneous or "internal consistency" or (cronbach* and (alpha or alphas)) or (cronbach* and (alpha or alphas)) or (item and (correlation* or selection* or reduction*)) or agreement or imprecision or "precise values" or test-retest or ((test and retest) or (reliab* and (test or retest))) or stability or interrater or inter-rater or intrarater or intra-rater or intertester or inter-tester or intratester or intra-tester or interobserver or inter-observer or intraobserver or intra-observer or intertechnician or inter-technician or interexaminer or inter-examiner or intraexaminer or intra-examiner or interassay or inter-assay or intraassay or intra-assay or interindividual or inter-individual or intraindividual or intra-individual or interparticipant or inter-participant or intraparticipant or intra-participant or kappa or kappa's or kappas or repeatab* or ((replicab* or repeated) and (measure or measures or findings or result or results or test or tests)) or generaliza* or generalisa* or concordance or (intraclass and correlation*) or discriminative or "known group" or "factor analysis" or "factor structure" or "factor structures" or dimension* or subscale* or (multitrait and scaling and (analysis or analyses)) or "item discriminant" or "interscale correlation*" or error or errors or "individual variability" or "interval variability" or "rate variability" or (variability and (analysis or values)) or (uncertainty and (measurement or measuring)) or "standard error of measurement" or sensitiv* or responsive* or (limit and detection) or "minimal detectable concentration" or interpretab* or ((minimal or minimally or clinical or clinically) and (important or significant or detectable) and (change or difference)) or (small* and (real or detectable) and (change or difference)) or "meaningful change" or "ceiling effect" or "floor effect" or "item response model" or IRT or Rasch or "differential item functioning" or DIF or "computer adaptive testing" or "item bank" or "cross-cultural equivalence")) not (addresses or biography or "case reports" or comment or directory or editorial or festschrift or interview or lectures or legal cases or legislation or letter or news or newspaper article or patient education handout or popular works or congresses or consensus development conference or practice guideline)).ab.

#### Scopus

( ( TITLE-ABS-KEY ( instrumentation  OR  methods  OR  "Validation Studies"  OR  "Comparative Study"  OR  "psychometrics"  OR  psychometr*  OR  clinimetr*  OR  clinometr* ) )  OR  ( TITLE-ABS-KEY ( "outcome assessment"  OR  "outcome assessment"  OR  "outcome measure*"  OR  "observer variation"  OR  "Health Status Indicators"  OR  "reproducibility of results"  OR  reproducib*  OR  "discriminant analysis"  OR  reliab*  OR  unreliab*  OR  valid*  OR  "coefficient of variation"  OR  coefficient  OR  homogeneity  OR  homogeneous  OR  "internal consistency" ) )  OR  ( TITLE-ABS-KEY ( cronbach*  AND  ( alpha  OR  alphas ) )  OR  ( cronbach*  AND  ( alpha  OR  alphas ) )  OR  ( item  AND  ( correlation*  OR  selection*  OR  reduction* ) )  OR  agreement  OR  imprecision  OR  "precise values"  OR  test-retest  OR  ( ( test  AND  retest )  OR  ( reliab*  AND  ( test  OR  retest ) ) ) )  OR  ( TITLE-ABS-KEY ( stability  OR  interrater  OR  inter-rater  OR  intrarater  OR  intra-rater  OR  intertester  OR  inter-tester  OR  intratester  OR  intra-tester  OR  interobserver  OR  inter-observer  OR  intraobserver  OR  intra-observer  OR  intertechnician  OR  inter-technician  OR  interexaminer  OR  inter-examiner  OR  intraexaminer  OR  intra-examiner  OR  interassay  OR  inter-assay  OR  intraassay  OR  intra-assay  OR  interindividual  OR  inter-individual  OR  intraindividual  OR  intra-individual  OR  interparticipant  OR  inter-participant  OR  intraparticipant  OR  intra-participant  OR  kappa*  OR  repeatab* ) )  OR  ( TITLE-ABS-KEY ( replicab*  OR  repeated )  AND  ( measure  OR  measures  OR  findings  OR  result  OR  results  OR  test  OR  tests ) )  OR  ( TITLE-ABS-KEY ( generaliza*  OR  generalisa*  OR  concordance  OR  intraclass  OR  correlation*  OR  discriminative  OR  "known group"  OR  "factor analysis"  OR  "factor structure"  OR  "factor structures"  OR  dimension*  OR  subscale*  OR  multitrait  OR  scaling  OR  analysis  OR  analyses  OR  "item discriminant"  OR  "interscale correlation*"  OR  error  OR  errors  OR  "individual variability"  OR  "interval variability"  OR  "rate variability" ) )  OR  ( TITLE-ABS-KEY ( variability  AND  ( analysis  OR  values ) ) )  OR  ( TITLE-ABS-KEY ( uncertainty  AND  ( measurement  OR  measuring ) ) )  OR  ( TITLE-ABS-KEY ( "standard error of measurement"  OR  sensitiv*  OR  responsive* ) )  OR  ( TITLE-ABS-KEY ( limit  AND  detection ) )  OR  ( TITLE-ABS-KEY ( "minimal detectable concentration"  OR  interpretab* ) )  OR  ( TITLE-ABS-KEY ( ( minimal  OR  minimally  OR  clinical  OR  clinically )  AND  ( important  OR  significant  OR  detectable )  AND  ( change  OR  difference ) ) )  OR  ( TITLE-ABS-KEY ( small*  AND  ( real  OR  detectable )  AND  ( change  OR  difference ) ) )  OR  ( TITLE-ABS-KEY ( "meaningful change"  OR  "ceiling effect"  OR  "floor effect"  OR  "item response model"  OR  irt  OR  rasch  OR  "differential item functioning"  OR  dif  OR  "computer adaptive testing"  OR  "item bank"  OR  "cross-cultural equivalence" ) ) )  AND  ( ( TITLE-ABS ( wellbeing  OR  "well being"  OR  well-being ) )  AND  ( TITLE-ABS ( employee  OR  employees  OR  worker  OR  workers  OR  staff  OR  personnel ) ) )
